# Implementing a Status-Neutral Approach to HIV in the Asia-Pacific

**DOI:** 10.1007/s11904-020-00516-z

**Published:** 2020-07-29

**Authors:** Nittaya Phanuphak, Reshmie Ramautarsing, Tanat Chinbunchorn, Rena Janamnuaysook, Supabhorn Pengnonyang, Krittaporn Termvanich, Pongthorn Chanlearn, Danai Linjongrat, Surang Janyam, Praphan Phanuphak

**Affiliations:** 1 Institute of HIV Research and Innovation, 319 Phayathai Road, Pathumwan, Bangkok, 10330 Thailand; 2 Mplus Foundation, 142 Chiang Mai Hod Road, Muang, Chiang Mai, 50200 Thailand; 3 Rainbow Sky Association of Thailand, 1 and 3 Ramkhamhaeng Road, Bangkapi, Bangkok, 10240 Thailand; 4 Service Workers in Group Foundation, Surawong Road, Bangrak, Bangkok, 10500 Thailand; 5 Thai Red Cross AIDS Research Centre, 104 Rajdumri Road, Pathumwan, Bangkok, 10330 Thailand

**Keywords:** Status-neutral, Asia-Pacific, HIV, Lay providers, Key population-led health services, Task shifting

## Abstract

**Purpose of Review:**

Globally, “undetectable equals untransmittable (U=U)” and “pre-exposure prophylaxis (PrEP)” have become crucial elements in HIV treatment and prevention programs. We reviewed the implementation of U=U and PrEP among countries in the Asia-Pacific region.

**Recent Findings:**

U=U and PrEP uptakes were limited and slow in the Asia-Pacific. Inadequate knowledge among health care practitioners and pervasive stigma towards individuals living with HIV and their sexual lives are key barriers for the integration of U=U into clinical practice. Paternalistic and hierarchical health care systems are major obstacles in PrEP implementation and scale-up. Countries with the most advanced PrEP implementation all use community-based, nurse-led, and key population-led service delivery models.

**Summary:**

To advance U=U and PrEP in the Asia-Pacific, strategies targeting changes to practice norm through wide-scale stakeholders’ training and education, making use of online health care professional influencers, and utilizing financial mechanism should be further explored through implementation research.

## Introduction

**Fig. 1:**
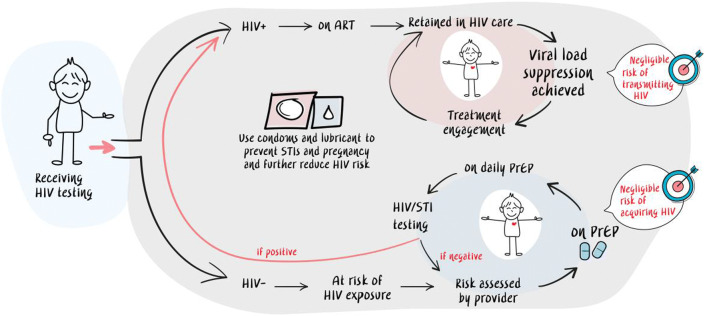
“Status-neutral” approach to HIV as a way to shift the messaging and programming paradigms of HIV treatment and prevention

“Undetectable equals untransmittable (U=U)” and “pre-exposure prophylaxis (PrEP)” have globally become crucial elements in HIV treatment and prevention programs over the past decade. Recently, a “status-neutral” approach to HIV (Fig. 1) has been proposed as a way to shift the messaging and programming paradigms of HIV treatment and prevention [1•]. The status-neutral approach begins with an HIV test which is followed by active engagement of that person regardless of their HIV status. Those who tested HIV-positive are engaged in treatment right away while those who tested HIV-negative are also immediately engaged in PrEP or post-exposure prophylaxis (PEP), visualizing that the clinical, programmatic, or social “HIV” divide is nonexistent. Both HIV-positive and HIV-negative individuals end at a common final stage of being continuously engaged in clinical care with negligible risk of either transmitting or acquiring HIV. Condoms are available to prevent sexually transmitted infections (STIs) and pregnancy regardless of HIV status.

The status-neutral approach may sound simple to understand and not be too challenging to implement in countries with an aim to end the HIV epidemic. However, implementing this approach will need mutual understanding and concerted commitments and efforts from the people, providers, and policy makers. We reviewed examples of the use of the status-neutral approach among countries in the Asia-Pacific region who have demonstrated some advancements in program implementation to address their HIV burden. We identified key common barriers in the region which need serious actions taken to break through in order to scale up this innovative approach.

## The Negligible Risk of Transmitting HIV: U=U Science and Concerns Among Health Care Practitioners

U=U science started two decades ago. In 2000, the Rakai study reported that sexual transmission risk of HIV increased with increasing HIV viral load, and they found no transmission when viral load was < 1500 copies/mL [2]. In 2008, the Swiss Statement was issued stating that “An HIV-positive individual not suffering from any other STIs and adhering to antiretroviral treatment (ART) with a completely suppressed viremia does not transmit HIV sexually” [3]. The statement faced a lot of controversies and scrutiny from researchers and practitioners at that time, as it was backed up by data from two observational studies and a mathematical model which were considered not strong enough [4]. It was until 2011, when the HPTN 052 study demonstrated 96% reduction in linked transmission in mainly heterosexual couples who were randomized to start ART early compared with those whose ART was delayed that “strong” evidence of U=U started to accumulate [5].

In 2014, the PARTNER 1 study, conducted in 14 countries in Europe, launched its preliminary data at the Conference on Retroviruses and Opportunistic Infections showing zero linked HIV transmission in heterosexual and gay serodiscordant couples with 44,400 condomless sex acts when viral load was < 200 copies/mL [6]. One caveat was that the number of gay couples was too small leading to the conduct of the PARTNER 2 study which only enrolled gay couples. In 2016, the PARTNER 1 study published their final result showing no HIV transmission with 58,000 condomless sex acts [7]. In that year, the HPTN 052 study reported sustained 100% preventive effect of ART after 5.5 years—with linked transmission only occurred when viral load was not undetectable due to the positive partner just starting ART or due to virologic failure [8]. The U=U campaign was launched that same year to bridge science to implementation for clients and communities [9].

In 2018, the Opposites Attract study, conducted only among gay couples in Australia, Brazil, and Thailand, added to the evidence that there was zero linked transmission among gay couples with 16,800 condomless sex acts when viral load was undetectable [10•]. And in 2019, the result from the PARTNER 2 study was published to confirm that the HIV transmission risk was zero among gay couples with 76,000 condomless sex acts if viral load was undetectable [11]. Altogether, the science behind U=U for HIV sexual transmission is very strong for both heterosexual and gay couples.

However, Australia is the only country in the Asia-Pacific where U=U has been publicly communicated and formally integrated in its National HIV Strategy [12]. Beyond that, only a few small U=U local events and dialogs have taken place [13–17]. There is an obvious lack of public statements from medical or public health authorities and community organizations to support U=U in the Asia-Pacific overall. U=U is merely seen as a topic for debate by health care practitioners during medical conferences, and not as a key topic to be actively advocated for by providers and communities of people living with HIV at a national level or a regional level. Even though Thailand was part of the HPTN 052 and the Opposites Attract studies, U=U has not yet been widely applied in clinical settings to empower people living with HIV to use ART to achieve and maintain their “untransmittable” status and live normal sexual and social lives. At the Thai Red Cross AIDS Research Centre, which is the largest HIV testing clinic in Southeast Asia located in Bangkok, U=U has been used as a key message in its same-day ART initiation service since 2017. High acceptance rates of same-day ART initiation service were seen at 92% of 1096 men who have sex with men (MSM), 94% of 68 transgender women, and 85% of 425 heterosexual men and women clients at this clinic [18]. ART was initiated on the same day of HIV diagnosis in 80% of MSM, 80% of transgender women, and 76% of heterosexual men and women in this cohort.

There has been no systematic study to evaluate the use of U=U to enhance HIV testing and treatment uptake and adherence in clinical settings or to address social and/or self-stigma related to people living with HIV in the Asia-Pacific. Uses of U=U message in clinics, if any, likely vary by health care practitioners’ trust in their clients’ ability to adhere to ART and attitude towards their clients’ sexual lives. From a few community forums and medical seminars in the region, it could be seen that health care practitioners are often reluctant to communicate the U=U message because they are afraid that individuals living with HIV would not be able to understand and maintain their undetectable status, especially if viral load is monitored just 1–2 times per year as is a common practice in the region. However, there is no evidence to support that more frequent viral load testing leads to a higher chance of maintaining viral load suppression. Furthermore, health care practitioners are very much concerned that individuals living with HIV will not be able to make a sensible judgment on when condoms are needed to prevent STIs or pregnancy, if use for prevention of HIV transmission is no longer needed. This may reflect the paternalistic view that practitioners in this region commonly have towards their patients [19, 20]. In addition, inadequate knowledge, research misinterpretation, and misconception around U=U are very common among the general public and health care practitioners, as recently demonstrated in Thailand. After a young gay man living with HIV posted an online message conveying that because of U=U, condomless sex is a safe-sex option for people living with HIV, public outrage blaming people living with HIV who have undetectable viral load and choose to have condomless sex as being totally irresponsible, and critiques from health care professionals questioning the science behind U=U emerged [21].

The incident in Thailand led the International AIDS Society, Asia Pacific Coalition on Male Sexual Health (APCOM) together with Prevention Access, Thai Ministry of Public Health with the WHO and the UNAIDS, and the principal investigators of the HPTN 052, Opposites Attract, and PARTNER studies to issue four public statements in February 2020 calling for public and medical communities to openly recognize, understand, and embrace U=U in their clinical practice and to challenge misinformation and break down stigma associated with HIV [22–25]. These statements, although scientifically clear and professionally strong, only interest stakeholders who were already advocates for U=U. Restating the strong science has not further affected clinical practice guidelines to change practice norm, as beliefs among health care practitioners about adverse consequences of U=U were likely not yet adequately addressed.

Several factors which could be associated with misperceptions around U=U, such as lifetime experience with HIV, attitude towards sexual behaviors and STIs, and current use of HIV testing and prevention services such as PrEP [26], should be explored among individuals living with and without HIV, as well as among health care practitioners in the Asia-Pacific. The information could help greatly in effectively tailoring strategies and points of strategic implementation to increase U=U understanding and decrease HIV-related stigma in specific groups of health care practitioners.

## The Negligible Risk of Acquiring HIV: PrEP Is Still “to Be” Scaled Up

If taken correctly, PrEP can reduce the risk of HIV acquisition to near-zero. As of January 2020, among 450,000–455,000 PrEP users globally, there were six cases of HIV seroconversion reported when high adherence to PrEP (as measured by PrEP drug concentration testing) could be confirmed [27, 28•]. Transmitted drug resistance to emtricitabine alone (two cases) or to both tenofovir disoproxil fumarate (TDF) and emtricitabine (three cases) was the likely cause of HIV seroconversion in five cases, although in two of them, missed acute HIV infection at PrEP initiation or acquisition during period of low adherence could not be clearly ruled out. The only one HIV seroconversion case without transmitted drug resistance reported high-inoculum effect with multiple exposures and concomitant lymphogranuloma venereum infection.

Among countries in the Asia-Pacific up to January 2020 [29], Australia has the highest number of PrEP users (26,000–27,000) followed by Thailand (10,500–11,500), Vietnam (5000–5500), New Zealand (2300–2800), Taiwan (1000–1500), and India (1000–1500). Other countries with small-scale demonstration projects of less than 1000 users include China, Laos, Japan, Malaysia, Philippines, and Nepal. Australia, Thailand, New Zealand, and Taiwan have a national level PrEP program.

New South Wales is the only geographic area in the Asia-Pacific where rapid, targeted, high-coverage roll-out of PrEP as part of combination HIV prevention approach to MSM through the Expanded PrEP Implementation in Communities in New South Wales (EPIC-NSW) study has resulted in a rapid decline in HIV diagnoses [30]. Data from the EPIC-NSW study has led the Australia Government to provide subsidized PrEP through its Pharmaceutical Benefits Scheme in April 2018. PrEP users are asked to co-pay at 40.3 Australian dollar (around 27.0 US dollar) for general patient rate or 6.5 Australian dollar (around 4.4 US dollar) for concessional patient rate for a 30-day supply of PrEP medication [12, 31]. To see the impact of PrEP roll-out on the reduction of new HIV cases, rapid and large-scale uptake is crucial [30]. The EPIC-NSW adopted a nurse-led PrEP provision model, coupled with a very strong community-led demand generation, in order to serve more than 8000 PrEP users through community-based sexual health clinics in under 2 years without additional resources [32•]. Efforts were made to have a legal instrument issued by the New South Wales Ministry of Health for the nurse-led PrEP provision model to be authorized. Extensive consultation with key stakeholders was crucial in addressing objection to the task shifting from pharmacists to nurses, mainly due to concerns around scope of practice and competency.

In Thailand, the National Health Security Office (NHSO) included PrEP as part of its health benefit package in October 2019 [33]. Thai PrEP users do not have to pay for the costs of PrEP medication or laboratory test under the NHSO scheme. In contrast to Australia, the roll-out of PrEP in Thailand has been slow with only 6.4% (9500 out of 148,500) of individuals, who are considered PrEP targets due to their behavioral risks, having already accessed PrEP over a 5-year period after PrEP was first recommended in its national guidelines in 2014 [34]. Almost half (48%) of Thai PrEP users by October 2019 received PrEP services at community-based organizations where PrEP is dispensed by key population lay providers [35•], one-third (34%) accessed fee-based PrEP, and the rest (18%) received PrEP from public hospitals. Key population (KP) lay providers deliver PrEP service as part of the key population-led health services (KPLHS) model which puts key populations at the center of the service. The KPLHS package is co-designed and co-delivered by key populations and health care professionals to ensure that services are client-centered and needs-based. KP lay providers are members of key populations themselves, and are trained and certified to provide HIV services in a clinical environment which is free of stigma, discrimination, and judgmental attitudes [37]. Being able to demonstrate the impact of KPLHS on HIV testing and PrEP uptake at the national level resulted in a series of intensive stakeholder meetings with professional institutions, and finally led Thai Ministry of Public Health to announce in June 2019 the legal endorsement to allow KP lay providers to provide HIV services in close collaboration with health care professionals as part of the national strategy to end HIV [38]. This legalization step has led stakeholders in Thailand to put collaborative efforts into increased domestic investments for KPLHS capacity building and financing, and to prepare to transition out from support by international agencies, to ensure KPLHS sustainability [39].

Vietnam started its PrEP pilot through the key population-led and -owned, social enterprise clinics in 2017 [40]. The national PrEP program was launched in 2018 in 11 provinces and was expanded to an additional 15 provinces in 2019. PrEP scale-up in Vietnam has been implemented using the differentiated service delivery concept to ensure that key populations who are in need of PrEP have access to diverse service options in both public and private sectors.

Almost all of these key PrEP programs in the Asia-Pacific use community-based or community-led services as the main service delivery sites, coupled with conventional health care facilities, in order to allow options to enhance access to PrEP service by as many individuals at risk for HIV acquisition as possible. The successful use of nurse-led and key population-led PrEP service delivery models in the Asia-Pacific highlights the importance of PrEP to be demedicalized [41]. Strategies to demedicalize PrEP must be considered seriously and should be tailored to target populations by every country who wants to rapidly implement and scale up PrEP.

## Paternalistic and Hierarchical Health Care Systems in the Asia-Pacific Have Delayed Advancement in Ending HIV Strategies

Health systems in Asia have long been dominated by the paternalistic view that health care providers and medical professionals have towards their patients [19, 20], as well as strong ownership and hierarchy of clinical roles authorized for each health care professional [20]. When this comes to HIV testing, the inability to control the pre- and post-test counseling process and the feared inability of users to follow the instruction for use of the HIV self-test kits worried practitioners to the level that the policy and law changes along with the registration of HIV self-test kits took more than a decade to happen in Thailand [42]. Nevertheless, Vietnam has successfully implemented both HIV self-testing and KP-led testing since 2016 to address the status of low HIV testing uptake among MSM [43]. HIV testing by trained lay providers, although with demonstrated effectiveness in increasing testing uptake among harder-to-reach and higher-risk key populations in a few countries in the Asia-Pacific [44, 45], has not yet been widely accepted by some health care professionals.

Moreover, strong and outdated professional ownership views also hinder the scale-up of HIV prevention and treatment programs, as it acts as key barriers for task shifting and task sharing which has long been recommended by the WHO [46]. For example, the success of ART and the elimination of mother-to-child transmission of HIV in Thailand [47, 48], relied heavily on the capacity of nurses to initiate and perform HIV testing, dispense antiretroviral drugs for treatment and prevention, and make efforts to ensure adherence to drugs and retention in services at a country-wide level. These efforts, however, have never been recognized officially and threats occurred from time to time from other professionals who felt that HIV testing and antiretroviral drug dispensing are not clinical roles authorized for nurses.

An effort to limit the responsibility to prescribe PrEP to those who are at high risk for HIV acquisition and are most in need for a comprehensive package of HIV prevention to “doctors” only, or even worse, to infectious disease doctors only, could delay PrEP scale-up in any country [32•, 35•, 49]. Not only is the conventional service delivery of PrEP by doctors generally inaccessible due to limited operating hours, which do not match with lifestyles of key populations who are the target of PrEP services, it is also not uncommon for PrEP services to be delivered in ART clinics, causing fear of stigma and discrimination pervasive in Asian society, creating another important barrier in accessing the service. Services are also unavailable due to a limited number of hospitals and doctors who agree to provide PrEP services [50] which could be due to already overwhelming ART clinics and other disease burdens and/or their ignorance about PrEP. In addition, many doctors could feel uncomfortable and reluctant providing PrEP service due to their lack of knowledge and skills in considering client’s contexts when assessing behavioral risk levels and offering PrEP in a way that fits well with each person’s lifestyle. The clinical complexity of PrEP prescription is small, but the social complexity of consideration of lifestyles and contextual aspects needed for taking care of PrEP users is large, and might be beyond the traditional skill set of many doctors.

In order for U=U to be fully integrated into clinical practice and for PrEP to be demedicalized for rapid scale-up, studies to explore people’s power and autonomy to make their health care decisions in the Asia-Pacific and its relationship to health care practitioners’ authoritarian roles should be conducted. Health care professional hierarchical power should also be addressed to challenge the status quo of task shifting and task sharing in most of the countries in this region.

## The Way Forward: Promising Implementation Strategies to Advance U=U and PrEP in the Asia-Pacific

Barriers to U=U implementation in the Asia-Pacific could possibly be explained by two psychological theory domains relevant to behavioral change of health care professionals [50]. These include (1) belief about consequences and (2) social influences. As described above, health care practitioners in the region believe that there are substantial risks that individuals living with HIV will stop using condoms to prevent STIs and/or pregnancy and may not be capable of keeping their undetectable and untransmittable status in the long term. In-depth explorations among health care practitioners to understand the foundation of beliefs about these adverse consequences, as well as to assess the correctness of knowledge and skills, of integrating U=U into their service are needed to understand current practice and identify opportunities for changes. With an obvious trend for health care practitioners nowadays to acquire their scientific knowledge through social media and online health care professional influencers, social norm establishment around U=U practice via this platform may be one potential strategy to persuasively communicate with practitioners, aiming to change their beliefs and behaviors.

Past and current PrEP guidelines, as well as campaigns to engage individuals who could benefit from PrEP, have focused on identifying people at risk and promoting PrEP as a risk reduction strategy—a so-called loss-framing message. Loss-framing messages focusing on risk can be effective for people who perceived themselves to be at risk [51]. However, recent studies have demonstrated common discordance between self-perceived risk and actual risk of HIV infection among MSM, transgender women, heterosexual cisgender women, and heterosexual and bisexual cisgender men in HIV testing and PrEP programs globally and in the region [52–56]. The use of gain-framing messages focusing on health promotion and being safe, rather than aiming at increasing risk perception, has recently been advocated to guide global PrEP roll-out [57]. The risk-based approach, which branded PrEP as a product for populations at high risk for HIV, not only affected potential users but also resulted in selective rather than inclusive prescribing of PrEP among practitioners. In addition, a rigid dosing and fixed clinic visit schedule commonly used in PrEP programs may inadvertently drive away users, causing low retention as conventionally measured. Since PrEP use can be flexible to match periods of different risk levels, monitoring “effective use” among PrEP users who are empowered to tailor their use of PrEP and frequency of clinic visit according to needs may allow programs to more realistically understand PrEP cascade [58]. Furthermore, financial reimbursement mechanism can potentially be used as an implementation strategy to reinforce inclusive PrEP prescription practice, regardless of clients being in particular populations, as currently being utilized by Thailand’s NHSO PrEP program. Recognizing the contribution of KPLHS in PrEP service expansion and the need for KPLHS sustainability, the NHSO domestic financing allows service reimbursement to both KP-led and facility-based PrEP programs. Investment in country-wide stakeholders’ training and education, used successfully for ART scale-up in the past, to ascertain understanding of gain-framing PrEP messages will likely be another key implementation strategy needed to ensure consistent messages across all sectors.

## Conclusion

The use of status-neutral approach to HIV, which highlights two equally important goals of achieving either negligible transmission status or negligible HIV acquisition status, is crucial in enhancing HIV testing, ART, and PrEP service uptake. The science of U=U and PrEP is strong and unquestionable. However, knowledge does not always translate into attitude and practice. Innovative strategies to enhance uptake of HIV testing, ART, and PrEP all depend on the ability of a person to take control over their health care decisions, and the ability of doctors to trust people to do so. Without identifying root causes and addressing barriers of our health care system, it will be unlikely for the countries in this region to be able to maximize the benefit of innovative HIV testing, ART, and PrEP strategies in the quest towards ending HIV by 2030. We would like to call for the following actions:

- Doctors and health care professionals in the Asia-Pacific need to have their knowledge updated and their applied skills built around innovative service delivery options for HIV testing, U=U, and PrEP.
- Doctors in the Asia-Pacific must realize that the paternalistic and hierarchical medical system has inadvertently been the key barrier to the scale-up of innovative strategies to end HIV. Implementation science research studies are needed to address this barrier through cultural, socioeconomic, gender, and political lenses in order to nurture task shifting and task sharing of clinical service roles to nurses, lay providers, and to the people.
- Social influences on U=U implementation through online health care professional influencers should be explored as one strategy to address common beliefs about adverse consequences of U=U and establish practice norm among health care practitioners in the Asia-Pacific.
- Gain-framing, inclusive, and community-led/based approach should guide PrEP service delivery implementation. Utilizing financial mechanism to reinforce this approach and investment in large-scale stakeholders’ training and education could be key strategies for rapid PrEP roll-out in the Asia-Pacific.
